# A Rare Occurrence of Bilateral Single-Rooted Mandibular First Molar

**DOI:** 10.1155/2022/1175630

**Published:** 2022-05-29

**Authors:** Urvashi Ujariya, Rajendra Bharatiya, Anjali Kothari, Viraj Shah, Mishri Parikh, Kruti Pandey, Dhara Mehta, Nishi Amin

**Affiliations:** Department of Conservative Dentistry, AMC Dental College and Hospital, Ahmedabad, Gujarat, India

## Abstract

It is essential to have an in-depth knowledge of root and root canal anatomy to prevent any iatrogenic errors. Many studies and case reports are present in the literature related to the anatomy of mandibular first molars, but most of them are on an extra number of roots and root canals. There are few studies related to a lower number of roots and root canals, but the occurrence of bilateral mandibular first and second molars with the presence of root fusion resulting in a single root is very rare. This case report presents the nonsurgical endodontic management of the left and right mandibular first molars with the presence of a single root confirmed using a CBCT and treated by placing an apical MTA plug followed by backfill using thermoplasticised gutta percha.

## 1. Introduction

A proper knowledge of root canal anatomy is necessary to plan and ensure a successful endodontic treatment. The mandibular first molars being the first permanent teeth to erupt often require endodontic treatment, as they are commonly affected by caries [1]. In most of the cases, mandibular first molars have two well-defined roots, one mesial and one distal, mesial root with two canals and distal with one or two canals [2, 3]. There are many studies in literature discussing the various variations of the mandibular first molars. These are not limited to the number of roots and root canals but also take into consideration the form and configuration of the root canals; studies of mandibular first molars with multiple canals, midmesial canal, middistal canal, radix entomolaris, paramolaris, and C-shaped canals have been reported [4–8].

However, along with the knowledge on multiple canals, it is crucial to be aware of the possibility of lesser number of roots and canals. Root fusion is one of the major reasons for a decrease in the number of roots and root canals. Root fusion is mainly formed by the combining of roots because of cementum deposition with time or when there is a failure of the Hertwig epithelial root sheath to develop or fuse in the furcation area. Different types of fusions have already been established for maxillary molars, but such is not recognized for mandibular molars. According to Hou et al., the prevalence of root fusion in mandibular second molars was found to be more than that in the mandibular first molars. Furthermore, in a similar study, he gave a classification of root fusion for mandibular molars as well [9]. Gopikrishna et al. have reported a case of a maxillary first molar with a single root and a single root canal [10]. Then, Krithikadatta et al. have reported a case of mandibular first molar with two roots and two root canals [11]. Thus, the aim of the study was to present a case report showing a rare canal variation to add to the body of knowledge and also discuss the similar relevant studies related to this anatomical variation.

## 2. Case Presentation

**Figure 1:**
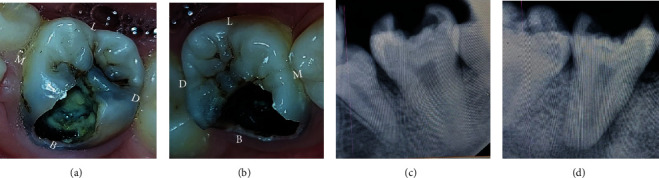
(a, b) Preoperative view of #36 and #46. (c, d) Preoperative IOPA of #36 and #46. B: buccal; L: lingual; D: distal; M: mesial.

**Figure 2:**
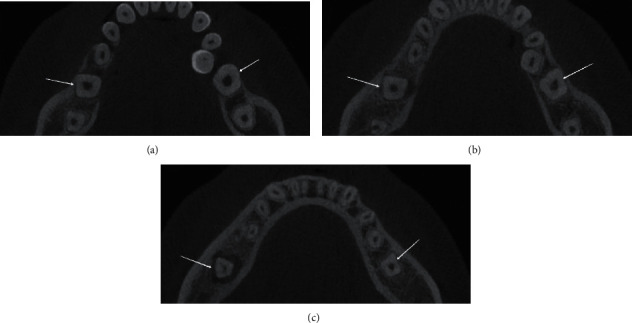
Axial section of CBCT scan showing the presence of a single canal in #36 and #46 at (a) cervical third, (b) middle third, and (c) apical third.

**Figure 3:**
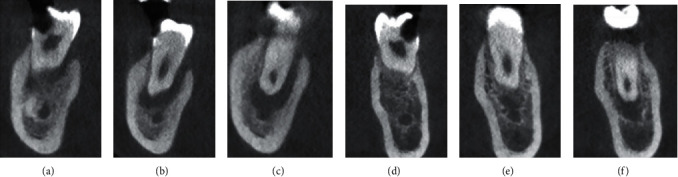
(a–c) Coronal section of #46 at cervical, middle, and apical thirds showing the presence of a single canal. (d–f) Coronal section of #36 at cervical, middle, and apical thirds showing the presence of a single canal.

A 17-year-old female patient reported to the Department of Conservative Dentistry and Endodontics with the chief complaint of pain and difficulty in chewing in the lower left back tooth region for the past two days. Past dental history revealed a painful response to cold temperature before two months. Her medical history was found to be noncontributory. Clinical examination revealed symptomatic teeth #36 and #46 with a deep carious lesion (Figures 1(a) and 1(b)). The tooth was tender on percussion. Thermal and electrical pulp testing elicited a negative response in the left mandibular first molar. The patient had an IOPA which revealed deep occlusal caries involving the buccal surface in close approximation to the pulp of the involved teeth and widening of the periodontal ligament space. IOPA also disclosed the unusual root anatomy of the mandibular first molar (Figures 1(c) and 1(d)). To ascertain the internal root canal anatomy of the teeth in a 3-dimensional (3D) manner, cone-beam computed tomography (CBCT) scan was scheduled (NewTom, Planmeca Oy, Helsinki, Finland). CBCT scan of the mandible was done with exposure parameters of 90 kV and 7 mA and a field of view (FOV) of 10∗5 cm and a voxel size of 0.08 mm. The images were reconstructed at 0.5 mm thickness increments. CBCT scans were viewed by two different observers for confirmation. From CBCT analysis, it was confirmed that teeth #36 and #46 revealed a single fused root with only one canal (Figures 2 and 3). A diagnosis of irreversible pulpitis with symptomatic apical periodontitis was made, and endodontic treatment was planned for teeth #36 and #46.

**Figure 4:**
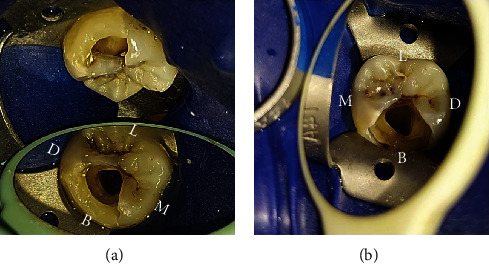
(a, b) Access openings of #36 and #46 showing the presence of a single canal.

The tooth was anaesthetized using 2% lidocaine with 1 : 80,000 adrenaline (Lignox, A 2% Indoco Remedies Ltd., Mumbai, India). Caries removal was done using a high-speed airotor under high-vacuum suction. The preendodontic buildup was performed on the compromised buccal surface using posterior composite resin (P60, 3M Dental Products., St Paul, MN, USA). After isolation with a rubber dam, the access cavity preparation was done using a round bur (Dentsply-Maillefer, Ballaigues, Switzerland) and a safe end carbide bur (Dentsply-Maillefer, Ballaigues, Switzerland). On examination, only one canal orifice was located, present centrally. Further inspection of the pulpal floor under a dental operating microscope revealed a lack of any other canal orifices (Figures 4(a) and 4(b)).

Canal patency was checked with #10 K-file (Dentsply-Maillefer, Ballaigues, Switzerland). The appointment was then concluded with the application of sterile cotton pellets and temporary restorations (Cavit, 3M ESPE AG, Seefeld, Germany).

In the next session, the correct working length was established using an electronic apex locator (Root ZX, Morita, Tokyo, Japan) and later confirmed using a radiograph. Biomechanical preparation was done using NiTi hand files. Since it was a very wide single canal, circumferential filing till #80K was done. 3% sodium hypochlorite solution (Prevest DenPro Limited, Jammu, India) was used as an irrigant during the whole instrumentation procedures. Instrumented canals were medicated with Ca [OH]2 (Prevest DenPro Limited, Jammu, India), and temporary dressing (Cavit,3M ESPE AG, Seefeld, Germany) was given. Radiographs were not possible after giving intracanal medications, since it clashed with COVID 2^nd^ wave and there were a few restrictions for taking the radiographs in the hospital.

**Figure 5:**
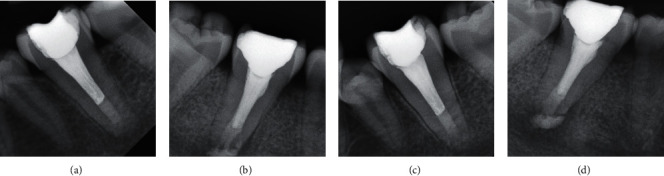
(a, b) Postobturation radiograph #36 and #46. (c, d) One-year follow-up radiograph #36 and #46.

After seven days, the patient was asymptomatic. 17% ethylenediaminetetraacetic acid (Pulpdent Corporation, Watertown, MA, USA) and 2% chlorhexidine digluconate (Prime Dental Products, Mumbai, India) were used as final irrigants. For obturation, it was planned to put an apical plug using MTA (Prevest DenPro Limited, Jammu, India) first followed by backfill using thermoplasticised gutta percha. This technique was selected because it ensures homogenous void-free obturation of the wide canal as well as to provide a better apical seal. Moreover, since there was a presence of a periapical lesion, MTA because of its bioinductive properties helps in accelerating osteogenesis. Four millimeters of apical MTA plug was placed. Appointment was concluded by placing a moist cotton pellet over the MTA to allow quicker setting, and a temporary dressing (Cavit, 3M ESPE AG, Seefeld, Germany) was placed over it. In the next session, backfill using thermoplasticised gutta percha (Ultrafil 3D, Coltene, USA) was done (Figures 5(a) and 5(b)). Finally, teeth were restored using permanent restorations (P60, 3M Dental Products, St Paul, MN, USA). After the completion of treatment in #36, similar treatment was performed for tooth #46 as well in three sessions. Pre-endo buildup was not done for #46 since proper isolation was obtained using rubber dam itself. The patient was recalled after a year for follow-up. She did not have any complaint of pain for both #36 and #46. Follow-up radiographs were also taken which showed good healing for both #36 and #46 and a decrease in the size of the periapical lesion for #46 (Figures 5(c) and 5(d)).

## 3. Discussion

This case presents the mandibular first molars having an unusual anatomy bilaterally with a single root and a root canal. This bilateral canal variation is very rare and approves the conclusion given by Sabala et al. that the rarer the variation is, the greater the possibility of it being bilateral [12]. The age of eruption for the mandibular first molar is six to seven years, and apical closure completes around eight to nine years of age. Changes in the normal root canal anatomy usually occur when any disturbances take place during the completion of canal differentiation about three to six years after root completion.

De Pablo et al. and Ballulaya et al. did a systematic review on the canal morphology of mandibular first molars, but they did not document this rare morphology in it [13, 14], whereas in Ruben et al.'s in vitro study, out of 125 mandibular molars of an Indian population, only one sample had a single root and root canal [15]. Ionnidis et al. in 2011 gave a case report on endodontic management of seven maxillary and mandibular molars with a single root and root canal requiring endodontic intervention [16]. Sooriaprakas et al. in 2014 also gave a case report on a similar case [17]. Similarly, Metgud et al. and Munnavali et al. also gave case reports on similar cases [18, 19]. A few details and remarks related to the above-mentioned case reports have been included in Table 1.

Root fusion in maxillary molars has been established, and many studies have been published for the same, but despite performing a comprehensive literature review, only one study by Hou et al. mentions the morphological classification of root fusion in mandibular molars. They stated in their study that root fusion in mandibular molars can be of three types, complete, buccal, or lingual fusion [9]. Concurring with the other studies, fusion was seen more in the mandibular second molars compared to the first. In our case, this was a type of complete fusion for both #36 and #46.

A CBCT was taken in this case to confirm this rare root canal morphology as advised by Koottor et al. and La et al. [20, 21]. The canal was located at the center of the chamber, and as it was already confirmed by CBCT for the presence of a single canal, no further search for another canal was done. Since the root canal space was large, proper removal of the entire pulp tissue and thorough debridement is necessary. Thus, the enlargement and preparation were done by circumferential filing technique using ISO taper files. Obturation is also a challenge because of this large pulp space; thus, after considering the possible options, four millimeters of apical plug using MTA was placed followed by backfill using thermoplasticised gutta percha. This technique was followed since it ensures a void-free obturation of the wide canal and provides a proper apical seal.

## 4. Conclusion

Root canal variations can also be in the form of root fusion presenting with fewer numbers of canals. CBCT is an important tool to ascertain the rare variations in such cases.

## Figures and Tables

**Table 1:** Case reports of mandibular first molars showing single canal.

Study	Tooth	Anatomical variation	Remarks
Metgud et al. [18]	36	Single conical root without any bifurcation	Taurodont with apical split
Ionnidis et al. [16]	36, 37, 47	Single root with a single root canal	Maxillary 16, 17, and 27 also showed the similar single root and root canal anatomy
Sooriaprakas et al. [17]	36	Single root with a single root canal	46 was extracted
Munnavali et al. [19]	46	Single root with a single root canal	47 also showed the similar single canal anatomy
Present study	36, 46	Single root with a single root canal	37 and 47 also showed the similar single canal anatomy
